# Neighborhood Walkability as a Predictor of Incident Hypertension in a National Cohort Study

**DOI:** 10.3389/fpubh.2021.611895

**Published:** 2021-02-01

**Authors:** Alana C. Jones, Ninad S. Chaudhary, Amit Patki, Virginia J. Howard, George Howard, Natalie Colabianchi, Suzanne E. Judd, Marguerite R. Irvin

**Affiliations:** ^1^Department of Epidemiology, University of Alabama at Birmingham, Birmingham, AL, United States; ^2^Department of Biostatistics, University of Alabama at Birmingham, Birmingham, AL, United States; ^3^School of Kinesiology, University of Michigan, Ann Arbor, MI, United States; ^4^Department of Biostatistics, School of Public Health (M.R.E.), University of Michigan, Ann Arbor, MI, United States

**Keywords:** walkability, built environment, hypertension, REGARDS, older adults, neighborhood walkability, neighborhood

## Abstract

The built environment (BE) has been associated with health outcomes in prior studies. Few have investigated the association between neighborhood walkability, a component of BE, and hypertension. We examined the association between neighborhood walkability and incident hypertension in the REasons for Geographic and Racial Differences in Stroke (REGARDS) Study. Walkability was measured using Street Smart Walk Score based on participants' residential information at baseline (collected between 2003 and 2007) and was dichotomized as more (score ≥70) and less (score <70) walkable. The primary outcome was incident hypertension defined at the second visit (collected between 2013 and 2017). We derived risk ratios (RR) using modified Poisson regression adjusting for age, race, sex, geographic region, income, alcohol use, smoking, exercise, BMI, dyslipidemia, diabetes, and baseline blood pressure (BP). We further stratified by race, age, and geographic region. Among 6,894 participants, 6.8% lived in more walkable areas and 38% (*N* = 2,515) had incident hypertension. In adjusted analysis, neighborhood walkability (Walk Score ≥70) was associated with a lower risk of incident hypertension (RR [95%CI]: 0.85[0.74, 0.98], *P* = 0.02), with similar but non-significant trends in race and age strata. In secondary analyses, living in a more walkable neighborhood was protective against being hypertensive at both study visits (OR [95%CI]: 0.70[0.59, 0.84], *P* < 0.001). Neighborhood walkability was associated with incident hypertension in the REGARDS cohort, with the relationship consistent across race groups. The results of this study suggest increased neighborhood walkability may be protective for high blood pressure in black and white adults from the general US population.

## Introduction

A primary risk factor for cardiovascular diseases (CVD), hypertension affects ~1 in three adults in the US (~75 million) (1). Regular physical activity (PA, ≥150 min per week) is associated with reduced risk of hypertension and is widely recommended for CVD prevention and all-cause mortality (2–4). Given the strong association between PA and health outcomes, there has been recent interest in how one's immediate surroundings (built environment (BE)) affect individual physical activity level. Neighborhood walkability, a measure of walking friendliness and important component of the BE, has been associated with PA and cardiometabolic risk factors in previous cohort studies (5–9). Particularly, there is a burgeoning interest in understanding the impact of neighborhood walkability on blood pressure and hypertension (10–14). Yet studies of walkability and hypertension in US populations are lacking, even though this association may differ by national and regional context. Moreover, in a cross-sectional analysis of neighborhood characteristics (including walkability) and prevalent hypertension in the US, Mujahid et al. noted that—because of the history of residential segregation—race may confound this relationship (15). Therefore, there is a need to the evaluate the association between walkability and hypertension and also consider the impact of racial and geographic differences.

The purpose of this study was to investigate the association between objectively measured neighborhood walkability and incidence of hypertension in the REasons for Geographic and Racial Differences in Stroke (REGARDS) Study. Given the protective effect of PA on hypertension and stroke risk, as well as the racial disparities in these cardiovascular outcomes, we sought to understand the relationship between walkability and new onset (incident) hypertension in a study population that includes both black and white participants from the continental US. In secondary analyses, we investigated effect modification by age, race, and geographic region and tested associations between walkability and hypertension status across two study visits.

## Materials and Methods

### Study Population

REGARDS is a population-study that was designed to observe racial and geographic differences in stroke incidence in the US, with oversampling in the Southeastern states with high stroke incidence, i.e., Stroke Belt and Stroke Buckle. The cohort is composed of 30,239 white and black adults aged 45 and older. Data was collected via telephone survey and in-home physical assessment at enrollment between 2003 and 2007 (baseline visit). Follow-up data were collected on ~51% of the original cohort (*n* = 15,550) using similar methods during a second visit an average of 10 years after enrollment (between 2013 and 2017). The REGARDS study design and objectives have been described elsewhere (16). The study protocol was approved by the institutional review boards of all participating institutions, and all participants provided written informed consent.

### Outcome of Interest

The measurement of blood pressure (BP) in the REGARDS study across both study visits has been described (17). Systolic blood pressure (SBP) and diastolic blood pressure (DBP) were defined as the average of two measurements taken by a trained technician using a standard protocol and regularly tested aneroid sphygmomanometer after the participant was seated for 5 min. Incident hypertension was defined as SBP ≥140 and/or DBP ≥90 mmHg and/or treatment with antihypertensive medication at the second study visit among those without hypertension at baseline. The use of anti-hypertensive medication was self-reported.

### Walkability Measurement

Street Smart Walk Score® is a validated and widely used walkability instrument that is derived from an algorithm that measures BE in proximity to each participant's residential address (18, 19). The score is based on proximity to walking routes to nearby amenities (e.g., parks, libraries, shopping centers, restaurants). The algorithm, after adjusting for intersection density and average block length, assigns the values from 0 to 100 with higher values reflecting greater walkability (0–49: Car-Dependent, 50–69: Somewhat Walkable, 70–89: Very Walkable, 90–100: Walker's Paradise). In the current study we dichotomized walk score as ≥70 (more walkable) and <70 (less walkable). All participants' addresses at baseline were validated and linked to a walk score. Scores were collected in 2018 as part of an ancillary study in REGARDS.

### Covariates

Covariates included baseline sociodemographic factors (age, sex, race, household income, geographic region), health-related behaviors (smoking, alcohol use, exercise frequency), and cardiometabolic traits (body mass index, dyslipidemia, diabetes). Age, sex, and race were self-reported. Race was classified as white or black. Income was self-reported and categorized into five groups: <$20,000, $20,000–$34,999, $35,000–$74,999, ≥$75,000, and refused to answer. Region was defined as Stroke Buckle (coastal plains of North Carolina, South Carolina and Georgia), Stroke Belt (the rest of North Carolina, South Carolina and Georgia, as well as Tennessee, Mississippi, Alabama, Louisiana, and Arkansas), or non-belt (other states of the continental US) (20). Self-reported smoking status was categorized as current smoking (yes or no). Self-reported alcohol use was categorized according to the National Institute on Alcohol Abuse and Alcoholism as heavy (>7 drinks/week for women, >14 drinks/week for men), moderate (≤7 drinks/week for women, ≤14 drinks/week for men), and none (0 drinks/week). Baseline exercise was categorized by self-reported frequency of exercise per week (none, 1 to 3, 4 or more). Body mass index (BMI) was calculated as a ratio of weight (kg) to square of height (m^2^). Dyslipidemia was defined as self-reported physician diagnosis of hyperlipidemia or current use of lipid-lowering medication. Diabetes was defined as self-reported current use of hypoglycemic medication, fasting blood glucose ≥126, or non-fasting blood glucose ≥200.

### Statistical Analysis

We compared subject characteristics and covariates between more walkable and less walkable areas using Pearson chi-square tests for categorical variables and independent *t*-tests for continuous variables among participants without baseline hypertension (see Table 1) and among all participants who completed a second visit (see Supplementary Table 1). In primary analyses (*N* = 6,894 without baseline hypertension), we determined overall and race-stratified risk ratios between walkability (dichotomized as <70 vs. ≥70 and, separately, considered as a continuous variable) and incident hypertension using modified Poisson regression with robust variance estimation. The risk ratios were unadjusted (Model 1, see Table 2) and adjusted for sociodemographic factors (age, sex, race, income, and geographic region), health-related behaviors (smoking status, alcohol use, and exercise frequency), cardiometabolic traits (diabetes, dyslipidemia, and BMI), and baseline blood pressure (SBP and DBP) in Model 2 (see Table 2). We further stratified these models by age (45–54, 55–64, ≥65) or geographic region (see Supplementary Table 2). We also conducted multivariate linear regression to determine the association between neighborhood walkability and second visit SBP and DBP, adjusting for covariates described above and antihypertensive medications to account for medication effect on blood pressure measurements (Table 3).

**Table 1:** Baseline characteristics of 6,894 REGARDS participants.

**Characteristic Mean (SD)/*N*(%)**	**Total**	**More walkable**	**Less walkable**	***P***
**N**	**6894**	**468**	**6,426**	
Age Mean (SD)	61.7 (8.4)	61.4 (8.1)	61.8 (8.4)	0.41
Females	3,771 (54.7%)	271 (57.9%)	3,500 (54.5%)	0.15
**Race**				**<0.0001**
White	5,087 (73.8%)	249 (53.2%)	4,838 (75.3%)	
Black	1,807 (26.2%)	219 (46.8%)	1,588(24.7%)	
**Education**				**0.022**
Less than high school	377 (5.5%)	23 (4.9%)	354 (5.5%)	
High school graduate	1,402 (20.3%)	81 (17.3%)	1,321 (20.6%)	
Some college	1,627 (26.1%)	92 (21.1%)	1,713 (26.5%)	
College graduate or above	3,310 (48.0%)	257 (54.9%)	3,053 (47.5%)	
**Income**				0.09
<$20,000	674 (9.8%)	61 13.0%)	613 (9.5%)	
$20,000–$34,000	1,292 (18.7%)	75 (16.0%)	1,217 (18.9%)	
$35,000–$74,000	2,407 (34.9%)	167 (35.7%)	2,240 (34.9%)	
$75,000 or more	1,763 (25.6%)	118 (25.2%)	1,645 (25.6%)	
Refused to Answer	758 (11.0%)	47(10.0%)	711 (11.1%)	
**Geographic Region**				**<0.0001**
Stroke Belt	2,278 (33.0%)	42 (9.0%)	2,236 (34.8%)	
Stroke Buckle	1,460 (21.2%)	27 (5.8%)	1,433 (22.3%)	
Non-Belt	3,156 (45.8%)	399 (85.3%)	2,757 (42.9%)	
Current Smoker	815 (11.8%)	59 (12.6%)	756 (11.8%)	0.59
Heavy Alcohol User (NIAAA)	298 (4.4%)	21 (4.6%)	277 (4.4%)	0.80
Exercise Frequency – None	1,788 (26.3%)	134 (28.8%)	1,654 (26.1%)	0.20
BMI Mean(SD)	27.8 (5.3)	27.6 (5.1)	27.8 (5.3)	0.41
Dyslipidemia	3,410 (51.2%)	213 (47.9%)	3,197 (51.4%)	0.15
Systolic Blood Pressure	118.7(11.2)	118.7(10.2)	118.7(11.3)	0.99
**Diastolic Blood Pressure**	73.6(7.6)	74.7(7.2)	73.5(7.7)	**0.002**
Diabetes	611 (9.1%)	50 (8.2%)	561 (9.0%)	0.13

*Boldface indicates statistical significance (p < 0.05) based on Pearson chi-square or independent t tests*.

**Table 2:** Risk ratios for incident hypertension for living in a more walkable neighborhood at baseline.

**Outcomes**	**Events/Total**	**Model 1^##^**	**Model 2^##^**
Incident Hypertension^a^			
Overall	2,515/6,894	**0.85,(0.74,0.98)**	**0.85,(0.74,0.98)**
Black	836/1,807	**0.80,(0.67,0.95)**	0.88,(0.74,1.06)
White	1,679/5,087	**0.77,(0.62,0.96)**	0.82,(0.66,1.02)

Results presented as

a *risk ratios (95% CI)*;

## *more walkable (Walk Score 70–100) compared to less walkable (<70, reference group); Model 1: Crude; Model 2: age, race, sex, region, income, alcohol use, smoking status, exercise, BMI, dyslipidemia, diabetes, baseline SBP, baseline DBP. Boldface indicates statistical significance (p <0.05)*.

**Table 3:** Association of walkability category with second visit SBP and second visit DBP.

**Outcomes**	**Model 1^##^**	***P***	**Model 2^##^**	***P***
Systolic Blood Pressure	β(SE)		β(SE)	
Overall	−0.82(0.65)	0.21	−0.38(0.67)	0.57
Black	−1.16(0.99)	0.24	0.16(1.06)	0.88
White	−1.39(0.88)	0.11	−0.64(0.89)	0.47
Diastolic Blood Pressure				
Overall	−0.65(0.41)	0.12	−0.52(0.43)	0.23
Black	–**1.52(0.63)**	**0.02**	−0.23(0.67)	0.74
White	−0.54(0.55)	0.33	−0.60(0.57)	0.29

*Results presented as beta estimates (SD); N = 6,894*;

## *more walkable (Walk Score 70–100) compared to less walkable (<70, reference group); Model 1: Crude; Model 2: age, race, sex, region, income, alcohol use, smoking status, exercise, BMI, dyslipidemia, diabetes, baseline SBP, baseline DBP, and second visit hypertensive medications. Boldface indicates statistical significance (p < 0.05)*.

Assuming that walkability at an individual level may remain relatively stable over time, we assessed the relationship between walkability and hypertension across two study visits (*N* = 15,550) in secondary analyses. We defined a multinomial outcome where participants who completed both study visits were categorized as hypertensive at both study visits (“always hypertensive”), normotensive at the first visit and hypertensive at the second visit (“incident hypertension”), hypertensive at the first visit and normotensive at the second visit (“blood pressure decline”), and reference category normotensive at both visits (“always normotensive”). We then conducted a multinomial logistic regression and report a crude and adjusted odds ratio for hypertension status across both visits considering age, race, sex, region, income, alcohol use, smoking status, exercise frequency, BMI, dyslipidemia, and diabetes as covariates (see Supplementary Table 3). All the analyses were performed using SAS v9.4 (SAS Corp).

## Results

**Figure 1:**
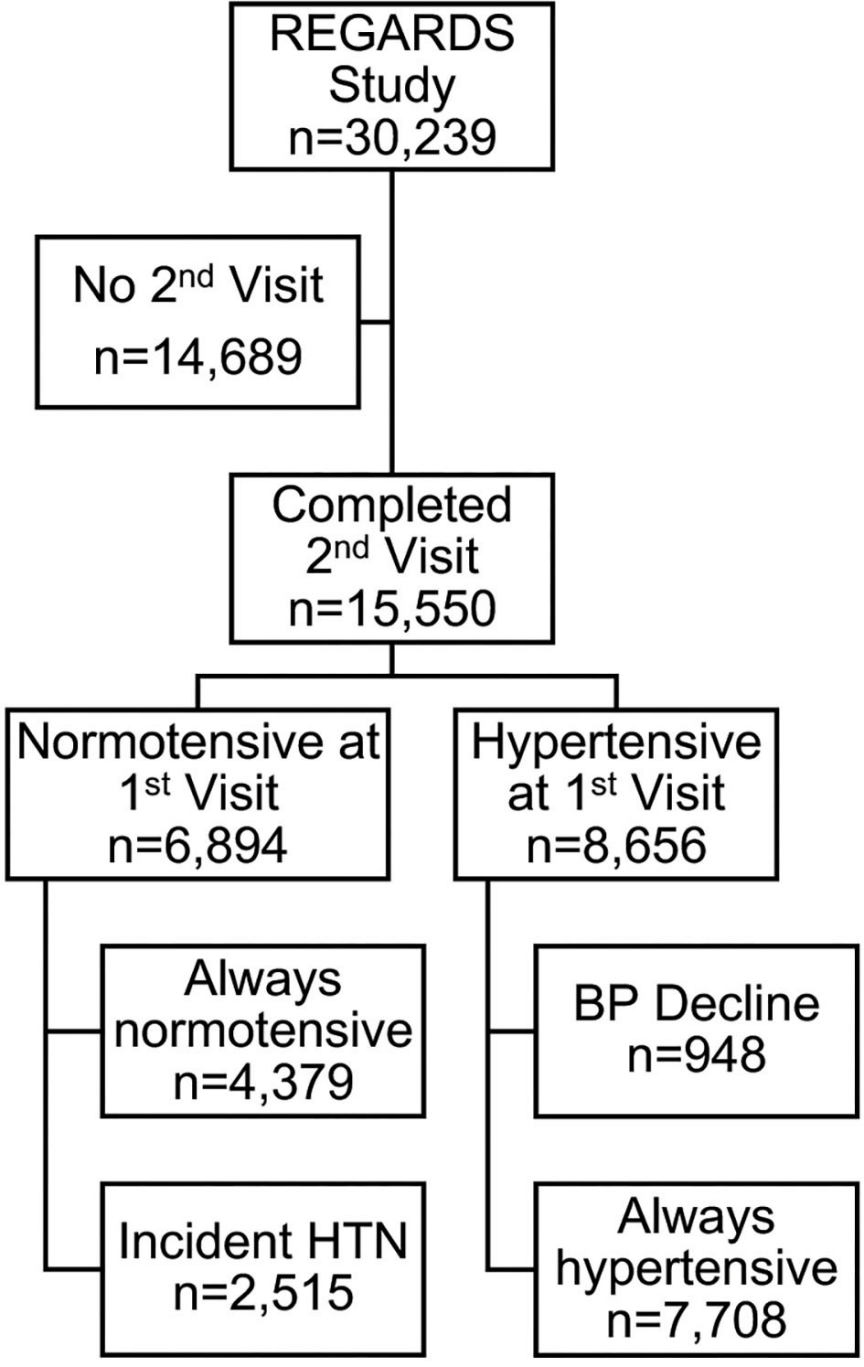
Exclusion cascade to identify REGARDS participants who were normotensive at baseline and later developed hypertension.

Among 6,894 participants without prevalent hypertension, 2,515 developed hypertension by the second visit (see Figure 1). A total of 6.8% (*N* = 468) were living in more walkable areas defined as walk score ≥70 at the first visit. Supplementary Figure 1 shows the distribution of walk scores among the 6,894 participants (mean(sd) 26.1(24.7), median 19). Those who lived in more walkable areas had mean(sd) age of 61.4(8.1) years, were more likely to be black and college graduates (see Table 1). Participants in more walkable areas were more likely living in non-belt areas compared to those in less walkable areas. Mean baseline SBP was similar in participants living in more walkable areas and those living in less walkable areas, whereas the mean DBP at baseline was slightly higher for those in more walkable areas. Baseline characteristics of the 15,500 participants who completed the two study visits (including participants with prevalent hypertension) can be found in Supplementary Table 1. Like the data presented in Table 1, those living in more walkable areas were more likely to be black, be college graduates, and live outside the stroke belt and stroke buckle.

The incidence of hypertension in those living in more walkable (≥70) areas at baseline was lower than those living in less walkable (<70) areas at baseline (31.4% vs. 36.9%, *P* = 0.02). As shown in Table 2, the higher risk of incident hypertension was resilient to covariate adjustment (Model 2, RR[95%CI]: 0.85[0.74,0.98]). There was not a significant interaction between race and walkability (*P* = 0.75). The results in the racial strata were consistent with that of the full cohort, although with the smaller race-specific sample sizes, these relationships did not remain significant after full adjustment in Model 2 (see Table 2).

After multivariable adjustment, walkability was not significantly associated with second visit SBP and DBP among those without prevalent hypertension (see Table 3). Diastolic blood pressure was lower among black participants living in more walkable areas compared to those in less walkable areas in crude analysis. This same trend was observed among whites although not significant. The relationships were not statistically significant after multivariable adjustment.

When stratifying the results by geographic region, the protective effect of higher neighborhood walkability for incident hypertension was consistent across strata (*P*_interaction_ = 0.69), but not statistically significant (see Supplementary Table 2). Participants in more walkable areas also had lower risk of incident hypertension across age categories with the strongest protective relationship observed in the youngest age group (45–54 years). However, we did not see modification of the relationship between neighborhood walkability and incident hypertension by age-group (*P*_interaction_ = 0.25), and the relationship was not significant in any age strata (see Supplementary Table 2).

In a secondary analysis of hypertension status across two study visits (*n* = 15,550, see Supplementary Table 3), living in more walkable areas was associated with a lower odds of being “always hypertensive” vs. “always normotensive” (OR [95% CI]: 0.70[0.59, 0.84]), and these associations persisted in both blacks and whites, separately. Additionally, the results for “incident hypertension” were consistent with the primary model. Finally, neighborhood walkability category was not associated with “blood pressure decline” vs. “always normotensive” as part of this analysis.

## Discussion

There is a growing interest in understanding the role of the BE in community member activity levels. Previous studies have linked neighborhood walkability to hypertension and blood pressure. We found in a population of geographically and racially diverse older adults that higher neighborhood walkability was protective for incident hypertension, even in the fully adjusted model including measures of exercise. The association was consistent in both race groups and was not modified by age and geographic region. Further investigation is needed to better understand these relationships and help spur additional investment in walking infrastructure which could help improve community health.

Previous work has asked similar questions on neighborhood walkability and incident hypertension (10–15). Multiple studies in Canadian cohorts have shown that living in a more walkable neighborhood (as measured by Walk Score and other validated walkability indices) predicts a lower risk of developing hypertension, diabetes, and CVD (10–12). Similarly, in a cross-sectional study of middle-aged and older adults in China, higher walkability was associated with lower odds of CVD. While exercise partially mediated this relationship, there was no significant interaction with BE (21). These findings, along with our significant results which remained after adjusting PA, suggest that there may be additional health-promoting factors in more walkable environments that benefit older adults even if they are less likely to be as physically active in those environments as they age.

Comparing these results to our own support the notion that neighborhood walkability may have a consistent protective effect across different sub-groups. However, it is important to note that these associations may differ depending on geography and/or age. For example, a study in an Australian cohort that was similar to REGARDS in terms of age and comorbidities found no significant association between neighborhood walkability (not measured by Walk Score) and incident hypertension (13). Similarly, a longitudinal study of older Taiwanese adults found no associations between Walk Score and exercise or hypertension (22). Yet in Portland, Oregon, higher neighborhood walkability was associated with lower blood pressure after 1 year of follow-up among adults aged 50–75 (20). In our stratified analyses, we found that higher neighborhood walkability was protective for incident hypertension among all regional and age groups (although not statistically significant in smaller strata), with greatest effects among those living outside the stroke belt/buckle and among younger age groups (<65). However, there were no significant interactions between Walk Score and age or region of the country related to stroke risk (belt, buckle, non-belt).

The REGARDS cohort is comprised of a biracial sample of older US individuals and we did not see modification of the relationship between walkability and incident hypertension by race. Additionally, we observed that walkability category was not only protective against incident hypertension among older normotensive adults, but also against persistent hypertension (i.e., “always hypertensive” at both study visits) which was consistent across race groups in a larger secondary analysis. These results could mean that walkability may not be a major contributor to racial disparities in hypertension risk, even though other neighborhood characteristics (e.g., socioeconomic status) have been linked to these disparities (23). These results suggest increased neighborhood walkability is an aspect of the BE that is consistently protective for hypertension across subgroups of older adults in the US.

### Strengths and Limitations

Overall, the mechanisms by which aspects of the BE, e.g., walkability, affect health outcomes is complex, and even the measurements we used are not without error. Street Smart Walk Score is limited in its ability to directly determine walking behaviors. The score calculation is based on the density of destinations in a given block, which does not necessarily lend itself to predicting whether people will walk (potentially due to neighborhood safety or effects of housing segregation) so much as it informs us that they are in close proximity to a place where they can walk (24–26). Even with these limitations, the score has been validated as an appropriate measurement of neighborhood walkability in the US (18, 19). In this study, we observed that neighborhood walkability was associated with hypertension risk among those in more walkable areas (Walk Score ≥70). However, in sensitivity analyses exploring other categorizations of the Walk Score— “Walkable” (≥50) vs. “Car-Dependent” (<50)—and as a continuous variable, there was not a significant association. These findings suggest that there may be a non-linear relationship between neighborhood walkability and hypertension, and future studies should continue to evaluate this relationship and potentially more sensitive definitions of walkability.

Although walkability data were based on participants' addresses at baseline (2003–2007), the Walk Scores were calculated in 2018 for a REGARDS ancillary study (5). The software did not allow us to backdate the calculation to agree with the timing of the baseline visit. Additionally, second visit data were collected an average of 10 years after the baseline visit. Therefore, we cannot fully account for how participants' environments changed during the time between the baseline visit and when the Walk Scores were collected (e.g., gentrification), as well as the second visit (e.g., moving to a different neighborhood). Finally, although we attempted to adjust for all potential confounders, including self-reported exercise, we could not rule out the potential for unknown or residual confounding (27). Still, we believe the size and nationwide, biracial composition of the cohort makes this study one of the most comprehensive studies of this walkability metric and incident hypertension to date.

In conclusion, we sought to determine if walkability is a novel risk factor for hypertension, and we found that neighborhood walkability may be protective for incident hypertension among older adults in a large sample from the REGARDS Study. Our study constitutes one of the first to report on the association between neighborhood walkability and incident hypertension in the US and evaluate potential effect modification by race, age, and geographic region. Future studies should address additional nuances in these relationships related to race, age, and region as well as other measures of the BE. Continued understanding of the relationship between the built environment and cardiovascular health could potentially lead to neighborhood improvements which spur cardiovascular disease risk reduction on the community level.

## Data Availability Statement

The datasets used and/or analyzed during the current study are not publicly available but are available from the corresponding author upon reasonable request.

## Ethics Statement

The studies involving human participants were reviewed and approved by University of Alabama-Birmingham Institutional Review Board. The patients/participants provided their written informed consent to participate in this study.

## Author Contributions

AJ initiated the study, performed the data analysis, and prepared the Introduction, Results, and Discussion sections of the text. NSC and AP assisted in the development and implementation of the analysis plan and prepared the Methods section of the text. VH and GH were instrumental in the data collection for the REGARDS cohort. GH also provided feedback on the data analysis plan. NC and SJ assisted in the study design, as well as provided the walkability data and their expertise on the metric in REGARDS. MI (corresponding) assisted in the study design, development of the analysis plan, and provided expertise on hypertension in REGARDS. Both SJ and MI provided mentoring and extensive feedback from the inception of this project to subsequent data analysis and manuscript preparation. They contributed equally as last authors. All authors contributed to the article and approved the submitted version.

## Conflict of Interest

The authors declare that the research was conducted in the absence of any commercial or financial relationships that could be construed as a potential conflict of interest.
