# Supplementary material for: Neighborhood Walkability as a Predictor of Incident Hypertension in a National Cohort Study
Source: Front Public Health. 2021 Feb 1;9:611895. doi: 10.3389/fpubh.2021.611895 (PMC7882902; doi:10.3389/fpubh.2021.611895)
Supplement: Supplementary file 1 [file Table_1.DOCX]

**Neighborhood Walkability as a Predictor of Incident Hypertension in a National Cohort Study**

Alana C Jones^1^ BA BS, Ninad S Chaudhary MBBS MPH^1^, Amit Patki MS^2^, Virginia J Howard PhD^1^, George Howard DrPH^2^, Natalie Colabianchi PhD^3,4^, Suzanne E Judd PhD^2*^, Marguerite R Irvin PhD^1*^

*Authors contributed equally as last authors

1 Department of Epidemiology, University of Alabama at Birmingham, Birmingham, AL

2 Department of Biostatistics, University of Alabama at Birmingham, Birmingham, AL

3 School of Kinesiology, University of Michigan, Ann Arbor, MI

4. Department of Biostatistics, School of Public Health (M.R.E.), University of Michigan, Ann Arbor, MI

Corresponding Author:

Marguerite Irvin

1665 University Blvd, Rm 220F

Birmingham AL, 35294

irvinr@uab.edu

T: (205) 975-7672, F: (205)-934-8665

Supplement: Figures: 1, Tables: 3

**Supplemental Figures/Tables**

Supplemental Figure 1. Distribution of Street Smart Walk Scores in Normotensive Participants. (n=6,894). Mean(SD): 26.11(24.69); Median: 19; IQR: 4-44

Supplemental Table 1. Baseline Characteristics of 15,550 REGARDS Participants Who Completed a Second Visit

Supplemental Table 2. Risk Ratios for Incident Hypertension for Living in a More Walkable Neighborhood, by Geographic Region and Age

Supplemental Table 3. Odds Ratios for Hypertension Status (N=15,550) when Living in a More Walkable Neighborhood at Baseline

| **Supplemental Table 1. Baseline Characteristics of 15,550 REGARDS Participants Who Completed a Second Visit** | | | | | |
| --- | --- | --- | --- | --- | --- |
| **Characteristic Mean (SD)/N(%)** | | **Total** | **More Walkable** | **Less Walkable** | **P** |
| **N** | | **15550** | **1037** | **14513** |  |
| **Age** Mean (SD) | | 63.2(8.4) | 63.4 (8.6) | 63.2 (8.4) | 0.48 |
| **Females** | | 8791 (56.5%) | 635 (61.2%) | 8156 (56.2%) | **<0.01** |
| **Race** | |  |  |  | **<0.001** |
|  | White | 9636 (62.0%) | 409 (39.4%) | 9227 (63.6%) |  |
|  | Black | 5914 (38.0%) | 628 (60.6%) | 5286 (36.4%) |  |
| **Education** | |  |  |  | **0.04** |
|  | Less than high school | 1267 (8.1%) | 95 (9.2%) | 1172 (8.1%) |  |
|  | High school graduate | 3703 (23.8%) | 231 (22.3%) | 3472 (23.9%) |  |
|  | Some college | 4149 (26.7%) | 248 (23.9%) | 3901 (26.9%) |  |
|  | College graduate or above | 6428 (41.3%) | 463 (44.6%) | 5965 (41.1%) |  |
| **Income** | |  |  |  | **<0.001** |
|  | Less than $20,000 | 2059 (13.2%) | 184 (17.7%) | 1875 (12.9%) |  |
|  | $20,000-$34,000 | 3467 (22.3%) | 223 (21.5%) | 3244 (22.4%) |  |
|  | $35,000-$74,000 | 5175 (33.3%) | 314 (30.3%) | 4861 (33.5%) |  |
|  | $75,000 or more | 3130 (20.1%) | 205 (19.8%) | 2925 (20.2%) |  |
|  | Refused to Answer | 1719 (11.1%) | 111 (10.7%) | 1608 (11.1%) |  |
| **Geographic Region** | |  |  |  | **<0.001** |
|  | Stroke Belt | 5238 (33.7%) | 85 (8.2%) | 5153 (35.5%) |  |
|  | Stroke Buckle | 3379 (21.7%) | 46 (4.4%) | 3333 (23.0%) |  |
|  | Non-Belt | 6933 (44.6%) | 906 (87.4%) | 6027 (41.5%) |  |
| **Current Smoker** | | 1774 (11.4%) | 131 (12.7%) | 1643 (11.4%) | 0.19 |
| **Heavy Alcohol User (NIAAA)** | | 655 (4.3%) | 43 (4.3%) | 612 (4.3%) | 0.97 |
| **Exercise Frequency - None** | | 4676 (30.4%) | 346 (33.8%) | 4330 (30.2%) | **0.02** |
| **BMI** Mean(SD) | | 29.4(6.0) | 29.4(6.0) | 29.4(6.0) | 0.96 |
| **Dyslipidemia – Yes** | | 8765 (58.2%) | 548 (55.6%) | 8217 (58.4%) | 0.09 |
| **Systolic Blood Pressure** | | 126.1(15.7) | 126.5(15.2) | 126.0(15.7) | 0.34 |
| **Diastolic Blood Pressure** | | 76.5(9.3) | 77.9(9.0) | 76.4(9.3) | **<0.001** |
| **Diabetes – Yes** | | 2673 (17.7%) | 190 (19.1%) | 2483 (17.6%) | 0.24 |
| Boldface indicates statistical significance (p<0.05) based on Pearson chi-square or independent t tests. | | | | | |

| **Supplemental Table 2. Risk Ratios for Incident Hypertension for Living in a More Walkable Neighborhood, by Geographic Region and Age** | | | | |
| --- | --- | --- | --- | --- |
| **Outcomes** | | **Events/Total** | **Model 1^##^** | **Model 2^##^** |
| **Region** | |  |  |  |
|  | *Stroke Belt* | 881/2278 | 0.92(0.61,1.39) | 0.90(0.59,1.38) |
|  | *Stroke Buckle* | 529/1460 | 0.82(0.45,1.46) | 0.97(0.58,1.60) |
|  | *Non-Belt* | 1105/3156 | 0.87(0.75,1.02) | 0.86(0.73,1.00) |
|  |  |  |  |  |
| **Age** | |  |  |  |
|  | *45-54* | 372/1270 | 0.70(0.45,1.09) | 0.70(0.43,1.11) |
|  | *55-64* | 1190/3156 | 0.83(0.68,1.00) | 0.84(0.69,1.03) |
|  | *65 and above* | 953/2468 | 0.95(0.76,1.17) | 0.91(0.73,1.13) |
| Results presented as ^a^ risk ratios (95%); ^##^ more walkable compared to less walkable (reference group); Model 1: Crude; Model 2: age, race, sex, income, alcohol use, smoking status, exercise, BMI, dyslipidemia, diabetes, baseline SBP, baseline DBP. Boldface indicates statistical significance (p<0.05). | | | | |

| Supplemental Table 3. Odds Ratios for Hypertension Status (N=15,550) when Living in a More Walkable Neighborhood at Baseline | | | | |
| --- | --- | --- | --- | --- |
| Categories | | **Counts** | **Crude ^##^** | **Adjusted^##^** |
| Always hypertensive | |  |  |  |
|  | *Overall* | 7708 | 0.90(0.78,1.04) | **0.70(0.59,0.84)** |
|  | *Black* | 3761 | **0.68(0.55,0.84)** | **0.72(0.56,0.92)** |
|  | *White* | 3947 | **0.61(0.49,0.77)** | **0.70(0.54,0.92)** |
| Incident hypertension | |  |  |  |
|  | *Overall* | 2515 | **0.78(0.64,0.96)** | **0.74(0.59,0.93)** |
|  | *Black* | 836 | **0.68(0.51,0.90)** | 0.76(0.55,1.06) |
|  | *White* | 1679 | **0.69(0.52,0.92)** | 0.74(0.54,1.01) |
| Blood pressure decline | |  |  |  |
|  | *Overall* | 948 | 0.82(0.62,1.10) | 0.80(0.57,1.11) |
|  | *Black* | 346 | **0.63(0.42,0.94)** | 0.68(0.42,1.09) |
|  | *White* | 602 | 0.79(0.52,1.20) | 0.97(0.61,1.54) |
| Always normotensive (reference) | |  |  |  |
|  | *Overall* | 4379 | - | - |
|  | *Black* | 971 | - | - |
|  | *White* | 3408 | - | - |
| Results presented as odds ratios (95%) for hypertension status;^##^ exposure more walkable compared to less walkable (reference); Models adjusted for age, race, sex, region, income, smoking status, alcohol use, exercise, BMI, dyslipidemia, and diabetes. Boldface indicates statistical significance (p<0.05). | | | | |
