# Supplementary figures and images for: Neighborhood Walkability as a Predictor of Incident Hypertension in a National Cohort Study
Source: Front Public Health. 2021 Feb 1;9:611895. doi: 10.3389/fpubh.2021.611895 (PMC7882902; doi:10.3389/fpubh.2021.611895)

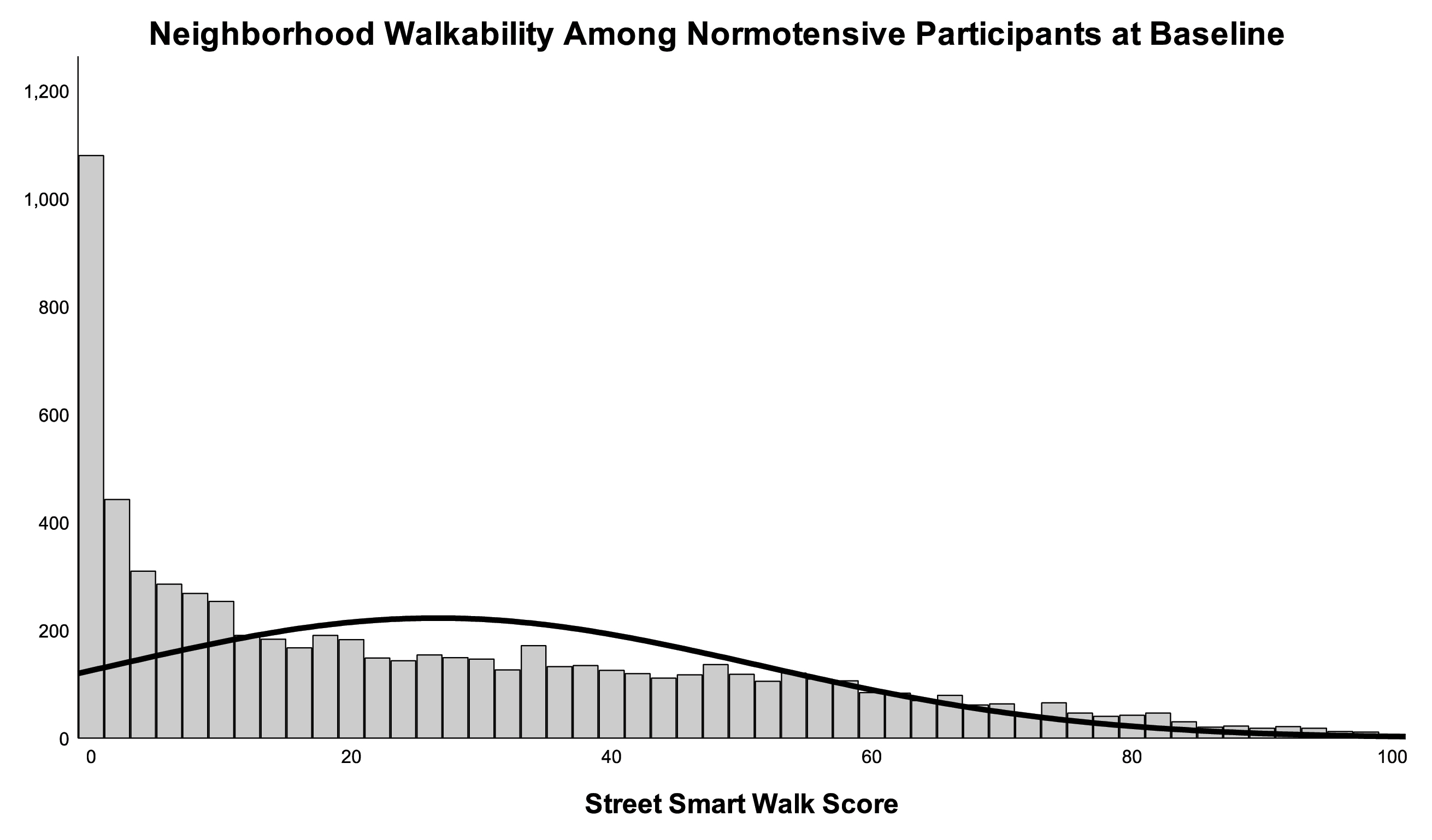

Supplement: Supplementary file 2 [file Image_1.JPEG]
